# Complementarity of prognosis models SAPS 3 and EuroSCORE in cardiac surgery

**DOI:** 10.1186/cc12412

**Published:** 2013-03-19

**Authors:** MJ Delgado-Amaya, E Curiel-Balsera, MD Arias-Verdu, E Castillo-Lorente, FJ Carrero-Gómez, E Aguayo-DeHoyos, A Herruzo-Avilés

**Affiliations:** 1 Hospital Regional Carlos Haya, Málaga, Spain; 2 Complejo Hospitalario de Jaén, Spain; 3 Puerta del Mar Hospital, Cádiz, Spain; 4 Virgen de las Nieves Hospital, Granada, Spain; 5 Virgen del Rocio Hospital, Seville, Spain

## Introduction

The aim of this study is to evaluate the performance of SAPS 3 and EuroSCORE and whether there is complementarity between them in ICU patients admitted after cardiac surgery.

## Methods

An observational, prospective and multicenter study of patients included in the ARIAM registry of adult cardiac surgery. We analyzed clinical variables, surgery data and postoperative complications, outcomes and scores (SAPS 3 and EuroSCORE). Discrimination was assessed using the area under the ROC curve. With the standardized mortality ratio (SMR) we evaluated the agreement between predicted and observed mortality. We used multiple logistic regression for multivariate analysis.

## Results

A total of 5,642 patients were included, admitted to any five hospitals, consecutively between 2008 and 2012. Mean age was 63.62 ± 12.80 years. Surgery room mortality was 1.4%, ICU mortality was 7.9% and in-hospital mortality was 11.2%. SAPS 3 was calculated in 5,383 patients. SAPS 3 was 40.91 ± 10.46, and the expected rate of mortality was 11.09% (with the southwestern Europe equation), with an observed hospital mortality of 9.6%, SMR = 0.87 (0.79 to 0.94). With the general equation, the expected mortality was 10.47%, SMR = 0.917 (0.843 to 0.996). Discrimination of SAPS 3 was evaluated using the area under the ROC curve, it was 0.77 (0.75 to 0.79). The 30-day mortality was 9.6%, with an expected 30-day mortality predicted by EuroSCORE of 7.86% (SMR = 1.22; CI = 1.12 to 1.32). The area under the ROC curve was 0.734 for EuroSCORE (0.712 to 0.755). Multivariate analysis with logistic regression showed complementarity between SAPS 3 and logistic EuroSCORE (vers. I). There is a relationship between hospital mortality and the probability of dying predicted by SAPS 3 (general equation), OR 1.05 (1.04 to 1.06), and logistic EuroSCORE (vers. I), OR 1.024 (1.015 to 1.032). The model with both variables has a similar discrimination to SAPS 3 alone. The area under the ROC curve of the combined model was 0.778 (0.758 to 0.799) and 0.77 (0.75 to 0.79) using SAPS 3 only.

## Conclusion

There is complementarity between the SAPS 3 model and EuroSCORE. However, a joint model with both prognosis scores has no appreciable improvement in discrimination, compared with the SAPS 3 model. In our study, SAPS 3 shows very useful to rule out that the superior observed mortality (compared with the mortality predicted by EuroSCORE) is not attributable to inadequate care of patients in the ICU, the observed mortality being even slightly below the predicted mortality for patients undergoing cardiac surgery at the reference hospitals in which the SAPS 3 system was developed.

